# Opinion, uptake and current practice of robot-assisted upper gastrointestinal and oesophagogastric surgery in the UK: AUGIS national survey results

**DOI:** 10.1308/rcsann.2024.0013

**Published:** 2024-03-06

**Authors:** P May-Miller, SR Markar, N Blencowe, JA Gossage, A Botros, PH Pucher

**Affiliations:** ^1^ Portsmouth Hospitals University NHS Trust, UK; ^2^ Oxford University Hospitals NHS Foundation Trust, UK; ^3^ University of Bristol, UK; ^4^ Guy’s and St Thomas’ NHS Foundation Trust, UK

**Keywords:** Robotic, Centres, Surgery, Survey

## Abstract

**Introduction:**

The uptake of upper gastrointestinal (GI) robotic surgery in the United Kingdom (UK), and Europe more widely, is expanding rapidly. This study aims to present a current snapshot of the practice and opinions of the upper GI community with reference to robotic surgery, with an emphasis on tertiary cancer (oesophagogastric) resection centres.

**Methods:**

An electronic survey was circulated to the UK upper GI surgical community via national mailing lists, social media and at an open-invitation conference on robotic upper GI surgery in January 2023. The survey included questions on surgeons' current practice or planned adoption (if any) of robotics at individual and unit level, and their opinions on robotic upper GI surgery in general. Priority ranking and Likert-scale response options were used.

**Results:**

In total, 81 respondents from 43 hospitals were included. Thirty-four resectional centres responded, including 30 of 31 (97%) recognised upper GI cancer centres in England. Respondents reported performing robotic surgery in 21 of 34 (61.8%) resectional centres, with a median of 65 procedures per centre performed at the time of the survey (range 0–500, interquartile range 93.75). Every centre without a robotic programme expressed a desire or had active plans to implement one. Respondents ranked surgeon ergonomics as the most important reason for pursuing robotics, followed by improvements in patient outcomes and oncological efficacy.

**Conclusions:**

Robotic upper GI practice is nascent but rapidly growing in the UK with plans for uptake in almost all tertiary centres. There is growing opinion that this is likely to become the predominant surgical approach in future with benefits to both patients and surgeons. This snapshot offers a point of reference to all stakeholders in upper GI surgery.

## Introduction

The uptake of robotic surgery in the United Kingdom (UK), and Europe more widely, is expanding rapidly. There was a fivefold increase in case volume in England recorded between 2013 and 2018 alone, with continuing exponential growth in case volume since.^1^ The purported advantages of a robotic surgical platform include better ergonomics with potential health benefits for surgeons, the immersive three-dimensional view, increased dexterity, the filtering of physiological tremor and a faster learning curve when compared with laparoscopic surgery.^2^^,^^3^ Studies have suggested that robotic surgery may be associated with improved patient outcomes, such as blood loss, length of stay and perioperative complications, with the majority of available evidence stemming from colorectal, urological or thoracic surgical domains.^3–5^

The uptake of robotics in the UK and Europe has lagged that of the US, in part because of cost. Despite this, it has expanded rapidly in the UK, driven in particular by initiatives such as the specialist commissioning of robotic prostatectomy by National Health Service (NHS) England.^6^ By comparison, the use of robotics in upper gastrointestinal (GI) surgery has experienced a much slower uptake, in part because of a weaker evidence base. Although there has been evidence of the safe use of robotic surgery in upper GI conditions for close to 20 years, it is only more recently that broader adoption within upper GI surgery has begun to take root.^7–9^

The introduction of new surgical techniques and technologies requires safe and graduated introduction, with corresponding safety and efficacy data, without which patient outcomes subjected to novel technologies may actually decline.^10^ These, in turn, colour surgeon opinion and interest in such technologies, which may be the ultimate arbiter of the speed and success of wider uptake – something that new technologies not infrequently fail to achieve, as with single-port or natural orifice surgery, for example.

The initial uptake of new technologies is most frequently driven by tertiary centres performing complex procedures with advanced resource availability. At the current inflection point, where the adoption of robotic surgery in UK upper GI surgical units is rapidly increasing pace, this study aims to present a current snapshot of the practice and opinions of the upper GI community with reference to robotic surgery, with an emphasis on oesophagogastric resection centres.

## Methods

The survey was developed by a core steering group under the auspices of the Association of Upper Gastrointestinal Surgeons of Great Britain and Ireland. Following initial piloting, an electronic survey was circulated to the UK upper GI surgical community, with an emphasis on oesophagogastric centres, via national mailing lists, social media and at an open-invitation conference on robotic upper GI surgery in January 2023 hosted by the AUGIS and the Royal College of Surgeons of England. The survey was closed on 31 January 2023.

The survey included questions on surgeons’ current practice or planned adoption (if any), with reference to robotic surgery, both at the individual and unit level, and surgeons’ opinions on robotic upper GI surgery in general. Priority ranking and Likert-scale response options were used. The survey explicitly targeted all upper GI surgeons, irrespective of whether they were currently involved or interested in robotics, to canvass a broad representative range of opinions.

Considering the purported surgeon ergonomic and health benefits with robotic platforms, the survey also included questions on musculoskeletal occupational health; these questions were adapted from the validated Nordic musculoskeletal questionnaire.^11^

### Statistical analysis

Results were collated using Excel. Differences in opinion between early (current robotic surgeons) and late/non-adopters (surgeons not currently practising robotics) were compared with the Mann–Whitney *U* test using SPSS^®^, with *p* < 0.05 deemed statistically significant. The results presented include all valid responses; invalid or incomplete responses were excluded, and thus reported denominators for some questions may not match overall total responses.

## Results

There were 81 respondents to the survey, representing 43 hospitals. The respondents included 66 consultants (male to female ratio 61:5), 13 trainees (9:4) and 2 non-training grade surgeons (both male). Of the respondents 47 of 81 (57%) were currently practising robotic surgery (early adopters), and 34 of 81 (43%) were not (late or non-adopters).

Forty-six of 78 (59%) respondents and 24 of the 43 (55.8%) represented respondent centres reported that they currently had access to robotic systems available for upper GI surgery. Of these, 23 hospitals had DaVinci systems (2 with DaVinci X, 15 DaVinci Xi and 6 with both), with one hospital using a Versius (CMR surgical) system at time of the survey.

The overall number of robotic upper GI procedures in the UK remains relatively low, with a median of 65 procedures per centre performed at the time of the survey (range 0–500, interquartile range [IQR] 93.75). With reference to robotic-assisted minimally invasive oesophagectomy, 20 respondents gave data on the number that have been performed in their unit, with a median of 32.5 cases (range 0–90, IQR 40.75).

### Unit-level adoption of robotics in oesophagogastric surgery

Survey responses included 34 oesophagogastric cancer centres: 30 of 31 (97%) recognised centres in England, and 3 further centres in Scotland and Wales. Of these 34 hospitals, 21 (61.8%) had current access to robotic surgery. Among hospitals not already performing robotic surgery, every centre reported plans to adopt robotics. Eight centres had a robot access plan agreed and were waiting to start their robotic programmes; one was in current negotiations with the trust and a further three had intentions to start and were either assembling a business plan or exploring their options.

Although all centres planned to progress to robotic cancer surgery, only 11 of the 34 (32%) respondent centres were currently performing resectional robotic surgery, of which only four were performing both abdominal and thoracic phases of Ivor Lewis oesophagectomy robotically. The remaining units were performing either only the abdominal phase (two centres) or thoracic phase (five) robotically.

Centres reported transitioning from minimally invasive oesophagectomy in six cases, and hybrid (laparoscopic abdomen, open chest) in three, with one centre moving from fully open and one from hybrid-open abdomen, thoracoscopic chest. For those performing robotic thoracic phase, eight units performed the oesophagogastric anastomosis in the same fashion as before transitioning to robotics (of which three used semi-mechanical linear stapled anastomosis and five used a circular stapled anastomosis: two with a transoral approach and three with transthoracic approach), compared with one unit who changed practice (from semi-mechanical linear stapled anastomosis to a circular stapled anastomosis with a transoral approach). A further two units had variation between surgeons in their anastomotic approach. One unit had two respondents who used the same approach (semi-mechanical linear stapled anastomosis) and one who changed from a circular stapled anastomosis to a linear stapled anastomosis. The other unit with variation had one respondent remain the same (hand-sewn) and one change from circular stapled with transthoracic approach to a hand-sewn anastomosis.

### Opinions and priorities

Calculating the mean priority ranking for the reasons for surgeons adopting (or wishing to adopt, for those not already practising) robotic surgery, improving surgeon ergonomics was ranked highest with a mean rank score of 3.11, followed by improvements in short-term patient outcomes (Table 1). Cost-effectiveness was ranked last with a mean rank score of 7.45. Overall priorities were similar for both those already practising robotics (early adopters) and those who were not (late/non-adopters).

**Table 1:** Priority ranked reasons for adopting robotic surgery

	Early adopters (current robotic surgeons)		Late adopters (non-robotic surgeons)	
	Rank	Mean rank	Rank	Mean rank
Ergonomics	1	3.11	1	2.90
Short-term outcomes	2	3.34	2	3.38
Improve radicality	3	3.87	6	5.48
Adopt/keep pace with modern technology	4	3.92	3	3.90
Long-term outcomes	5	4.45	4	4.79
Unit prestige	6	6.05	8	6.10
Efficiency	7	6.39	7	6.03
Keep pace with other units	8	6.42	5	4.97
Cost-effectiveness	9	7.45	9	7.45

**Figure 1:**
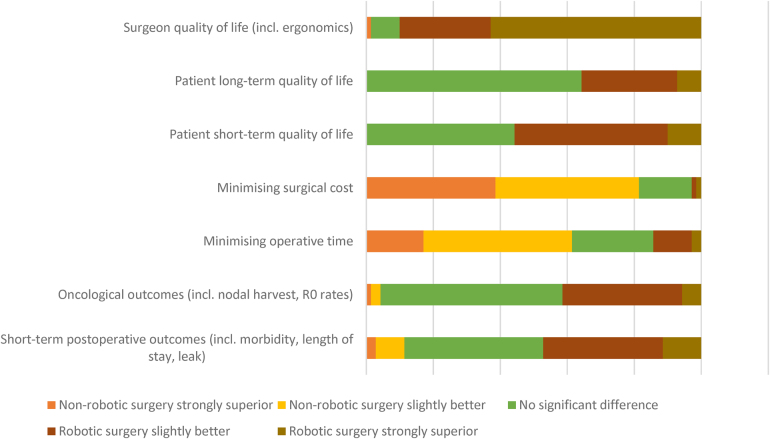
Likert-scale responses showing opinions on robotic surgery compared with non-robotic surgery across domains

Surgeon opinions on how robotic surgery compared with non-robotic approaches are detailed in Figure 1. The summary opinion was that robotic surgery represented a superior approach in all areas other than cost and operative time when compared with non-robotic approaches, with the balance of opinion strongest for surgeon quality-of-life advantages, followed by short-term patient quality-of-life. There were no statistically significant differences between the responses of early and late adopters.

**Figure 2:**
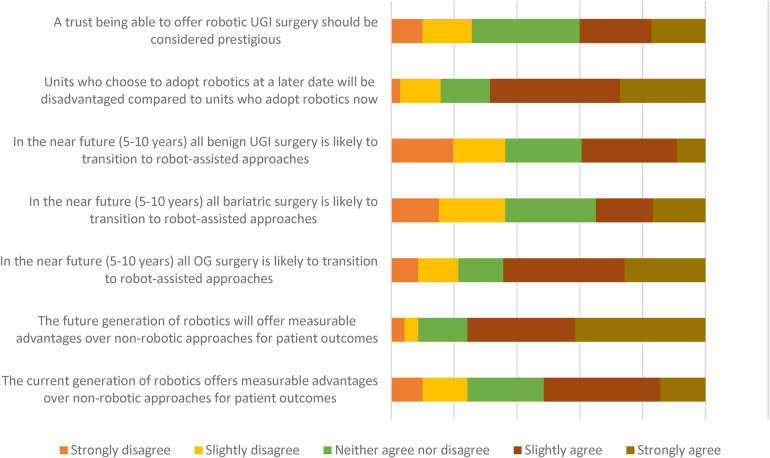
Likert-scale responses regarding the current and future impact of robotic surgery

Regarding the future of robotic surgery (Figure 2), the majority of respondents felt that robotic surgery would continue to be adopted, and that the strongest anticipated uptake was likely to be in oesophagogastric surgery, with less priority for bariatric or benign upper GI procedures. Very strong agreement was seen on the likelihood of future generations of robotic platforms offering increased or measurable advantages for patient outcomes. Perhaps unsurprisingly, the only question wherein a statistically significant difference between early and late/non-adopter respondents was seen was the question: “the current generation of robotics offers measurable advantages over non-robotic approaches for patient outcomes”, with a more positive response in the early adopter group (mean Likert response 2.87 vs 3.67, *p* = 0.009).

### Surgeon ergonomics

A significant majority of surgeons reported regularly experiencing at least some recent musculoskeletal pain (78.6% in the past 12 months); 21% reported that they had been unable to carry out normal activities at some point in the past 12 months as a result (Table 2).

**Table 2:** Results of reported musculoskeletal pain (modified Nordic questionnaire)

Have you at any time during the last 7 days suffered musculoskeletal pain or discomfort (neck, back, shoulders, arms, hips, legs)?	37/70 (52.9%)
Have you at any time during the last 12 months suffered musculoskeletal pain or discomfort (neck, back, shoulders, arms, hips, legs)?	55/70 (78.6%)
Have you at any time during the last 12 months taken any opiate-based analgesia (including tramadol, codeine or any other opiates) for relief of musculoskeletal pain or discomfort (neck, back, shoulders, arms, hips, legs)?	11/70 (15.7%)
Have you at any time during the last 12 months been prevented from carrying out normal activities owing to musculoskeletal pain or discomfort (neck, back, shoulders, arms, hips, legs)?	15/70 (21.4%)
Have you at any time during the last 12 months been forced to take any time off work owing to musculoskeletal pain or discomfort (neck, back, shoulders, arms, hips, legs)?	5/70 (7.1%)

## Discussion

Robotic upper GI surgery in the UK and Europe is nascent but rapidly accelerating. We present a snapshot audit, reporting on the current state of practice and opinions on robotic upper GI surgery in the UK. The results show that robotic surgery is widely expected to continue to expand, especially in the area of oesophagogastric surgery. Surgeons broadly believe that robotic surgery can improve outcomes for patients and reduce health problems for surgeons. Despite some individual respondents voicing neutral or negative opinions with reference to robotics, every responding hospital has either procured access to a robotic platform, or explored the possibility of doing so.

Not all hospitals have the resources or volume to accommodate a robotic system; however, the accelerating expansion of robotics means that it cannot be ignored. This survey highlights the perceived prestige afforded units with a robotic service, and that early adopters are perceived as possibly enjoying benefits compared with late adopter organisations – these may include surgical learning curves, establishing procurement pathways and promoting staff recruitment/retention. Robotic platforms may also have additional benefits for surgeon ergonomics and health; the longer term impact that this may have on surgeons, health systems and health economics is the subject of research but has yet to be definitively elucidated.

It should be recognised, however, that robotic surgery is no panacea. Respondents believe that currently robotic surgery is more expensive and may result in longer operative times. However, evidence suggests that once the significant learning curves – for both surgeons and surgical theatre teams – are overcome, operative times for robotic surgery may be equivalent or even shorter than for laparoscopic surgery.^12,13^ Costs are expected to continue to reduce, particularly with the introduction of new competitors into the surgical robotics market, an industry that has been dominated by an effective monopoly over the past 20 years.

The high incidence of musculoskeletal problems is consistent with the other published data and appears to affect surgeons who practise both open and laparoscopic surgery.^14^ There are data indicating that robotic surgery shows low-risk ergonomic stress in robotic vs medium-risk ergonomic stress in laparoscopic surgeons performing bariatric surgery.^15^ This survey demonstrates that surgeon ergonomics is a significant factor influencing the surgeons’ decisions on the adoption of a robotic system in their unit.

The effectiveness of robotic upper GI surgery continues to be the subject of research and debate, with limited contemporaneous high-quality evidence available. The available evidence has suggested that robotics has reached a stage where this approach can now translate into meaningful and measurable benefits for patients, and further studies are ongoing.^16–18^

### Study limitations

The results presented here should be considered within the limitations of any voluntary survey, particularly with reference to respondent self-selection bias. Because the survey was circulated openly via multiple open advertising platforms, the response rate cannot be ascertained. Forty-three respondent hospitals and 81 surgeons capture only a fraction of the total number of hospitals and upper GI surgeons across the UK. Self-selection response bias means that robotic surgeons made up the majority of respondents, despite explicit efforts to include those not involved in robotics. However, by including responses from all but one of the tertiary resection centres in England, we believe this survey accurately paints a picture of current practice at the unit level in oesophagogastric centres, from which advances in surgical technology are likely to diffuse to less-specialist centres in future. Anecdotally, since completion of this survey, an increasing number of non-tertiary centres have begun to adopt upper GI robotics for benign and bariatric surgery.

## Conclusions

Surgical robotics continues to be adopted at pace in the UK and elsewhere. Where this was perhaps once considered a niche practice, there is growing opinion, as reflected in this study, that this is likely to become the predominant surgical approach in future and may offer measurable benefits to both patients and surgeons. This snapshot offers a point of reference to all stakeholders in upper GI surgery.
